# Supplementary material for: Molecular Dynamics Study of α-Synuclein Domain Deletion Mutant Monomers
Source: Biomolecules. 2025 Nov 10;15(11):1577. doi: 10.3390/biom15111577 (PMC12650653; doi:10.3390/biom15111577)
Supplement: Supplementary file 1 [file biomolecules-15-01577-s001.zip › biomolecules-3463522-supplementary.pdf]

Molecular Dynamics Study of α-Synuclein Domain Deletion Mutant Monomers: Supplementary Information

*Noriyo Onishi, Nicodemo Mazzaferro, Špela Kunstelj, Daisy A. Alvarado, Anna M. Muller, Frank X. Vázquez**

Department of Chemistry, St. John's University, Queens, NY 11439, *USA*

*Author to whom correspondence should be addressed vazquef1@stjohns.edu

The sequence of the protein α-synuclein (aSyn) includes the consensus repeat motif KTKEGV. The location of these motifs and their sequences is shown in Table S1.

Sequence	Residue Number
KAKEGV	10-15
KTKQGV	21-26
KTKEGV	32-37
KTKEGV	43-48
KTKEQV	58-63
KTVEGA	80-85

Table S1. Location and sequence of consensus KTKEGV repeat motifs in aSyn.

Fully extended, straight chain proteins were used as an initial starting structure so that there would be no structural bias introduced into our simulations. These fully extended structures were then collapsed using *in vacuo* energy minimization and molecular dynamics simulations. The initial elongated structure and the resulting collapsed structure for the WT aSyn protein is shown in figure S1.

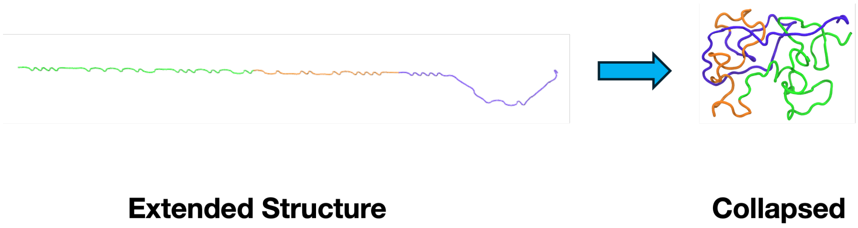

Figure S1. Example of an elongated protein and the resulting collapsed structure. The proteins are shown using the New Cartoon representation, with the N-terminal domain shown in green, the NAC domain in orange, and the C-terminal domain in violet.

The fractions for each cluster are shown in Table S2; however, it is important to note that because we did not use the GAMD reweighting scheme, that these frequencies are not the absolute values, even though their relative ranking should be correct.

Cluster	WT	ΔN	ΔC	isoNAC
**1**	0.228	0.209	0.219	0.127
**2**	0.185	0.140	0.205	0.119
**3**	0.178	0.125	0.118	0.114
**4**	0.126	0.114	0.091	0.112
**5**	0.066	0.114	0.087	0.109
**6**	0.055	0.093	0.080	0.100
**7**	0.051	0.088	0.076	0.096
**8**	0.049	0.051	0.056	0.087
**9**	0.032	0.049	0.052	0.087
**10**	0.030	0.017	0.017	0.050

Table S2. Fractions for each k-means cluster.

The center structures for each of the top ten clusters of the isoNAC variant were aligned with respect to the trace and are shown in Figure S1. The overlaid structures show how the NAC domain mostly adopted compact structures when not in contact with the flanking domains.

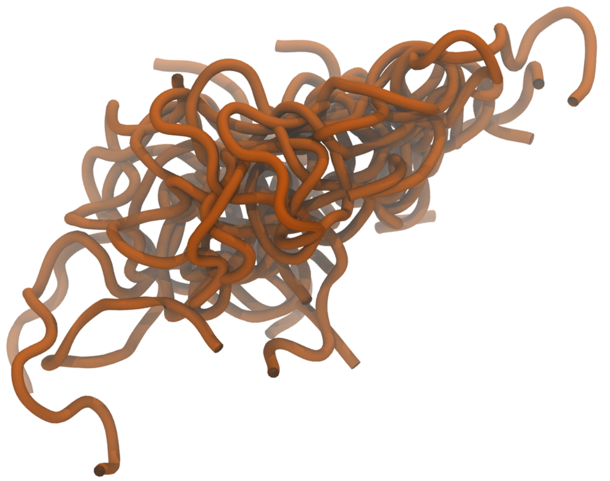

Figure S2. The center structures for each of the top ten clusters of the isoNAC variant. The proteins are shown using the New Cartoon representation.

The individual contact maps for the ΔN and ΔC protein variants are shown in Figure S2. The maps show the most probable distance between any pair of residues. Only values up to 12 Å are shown to highlight the contacts.

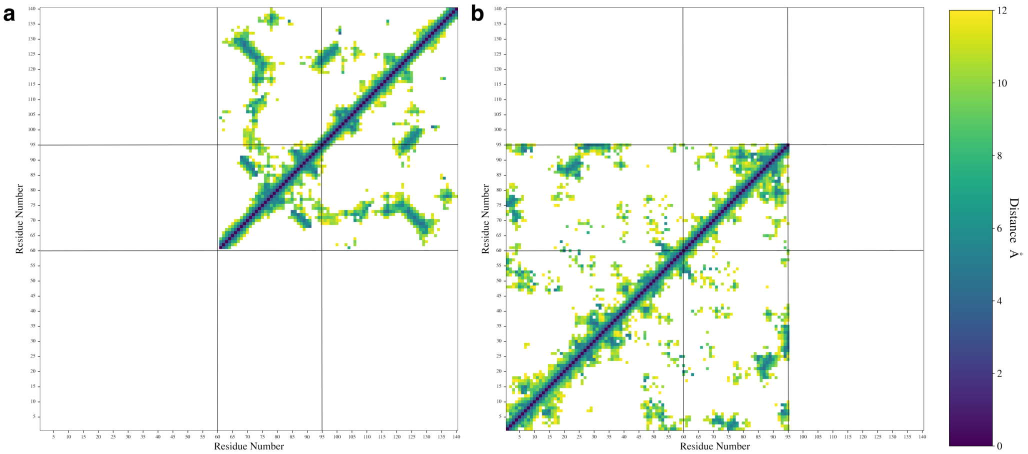

Figure S3. The individual contact maps of the most probable distance between amino acid pairs for the ΔN (a), and ΔC (b) variants.

The radius of gyration, R_g, of the entire protein is shown for each variant in Figure S3. The R_g generally shows the expected scaling trend, with the smaller isoNAC variant having sampled smaller values and the longer full-length WT protein larger R_g values. The ΔN and ΔC variants, even though their length differs by 15 residues, had similar R_g distributions.

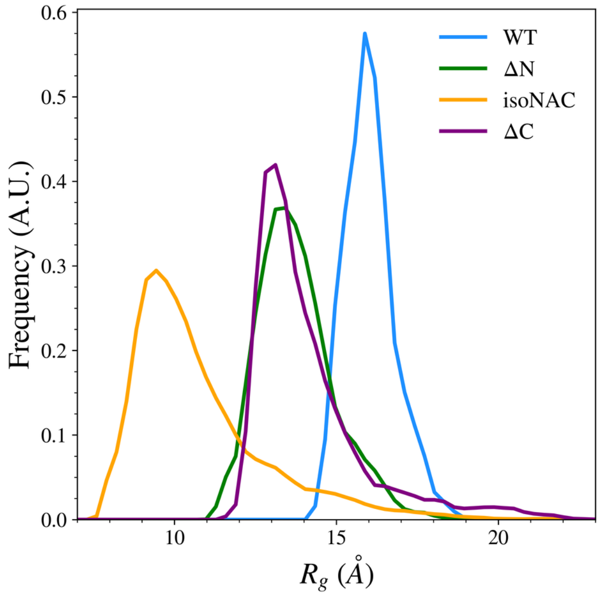

Figure S4. The radius of gyration, R_g, of the full protein for all simulated variants.
